# The COVID-19 pandemic and changes in social behavior: Protective face masks reduce deliberate social distancing preferences while leaving automatic avoidance behavior unaffected

**DOI:** 10.1186/s41235-023-00528-4

**Published:** 2024-01-08

**Authors:** Esther K. Diekhof, Laura Deinert, Judith K. Keller, Juliane Degner

**Affiliations:** 1 Department of Biology, Neuroendocrinology and Human Biology Unit, Faculty of Mathematics, Informatics and Natural Sciences, Institute for Animal Cell and Systems Biology, Universität Hamburg, Martin-Luther-King-Platz 3, 20146 Hamburg, Germany; 2 Department of Psychology, Universität Hamburg, Hamburg, Germany

**Keywords:** Social distancing, Automatic behavior, Deliberate behavioral motive, Approach-avoidance behavior, Coronavirus pandemic, Protective face mask, VAAST

## Abstract

Protective face masks were one of the central measures to counteract viral transmission in the COVID-19 pandemic. Prior research indicates that face masks impact various aspects of social cognition, such as emotion recognition and social evaluation. Whether protective masks also influence social avoidance behavior is less clear. Our project assessed direct and indirect measures of social avoidance tendencies towards masked and unmasked faces in two experiments with 311 participants during the first half of 2021. Two interventions were used in half of the participants from each sample (Experiment 1: protective face masks; Experiment 2: a disease prime video) to decrease or increase the salience of the immediate contagion threat. In the direct social avoidance measure, which asked for the deliberate decision to approach or avoid a person in a hypothetical social encounter, participants showed an increased willingness to approach masked as opposed to unmasked faces across experiments. This effect was further related to interindividual differences in pandemic threat perception in both samples. In the indirect measure, which assessed automatic social approach and avoidance tendencies, we neither observed an approach advantage towards masked faces nor an avoidance advantage for unmasked faces. Thus, while the absence of protective face masks may have led to increased deliberate social avoidance during the pandemic, no such effect was observed on automatic regulation of behavior, thus indicating the relative robustness of this latter behavior against changes in superordinate social norms.

**Supplementary Information:**

The online version contains supplementary material available at 10.1186/s41235-023-00528-4.

## Introduction

Protective face masks have been one of the central measures in the COVID-19 pandemic to limit the spread of the highly transmissible coronavirus (Li et al., 2020). In Germany, face masks became an everyday companion during the pandemic, and wearing them in public places such as supermarkets and public transport was mandatory in 2021, when the data collection for this study took place (Bundesregierung, 2020, 2021a, 2021b, 2023).

Many public debates during and after the Pandemic included the assumption that both the enhanced perceived infection risk as well as the use of protective masks may affect social cognition and interaction behavior. While a perceived contagion threat may provoke general social distancing and avoidance tendencies, this may only apply to unmasked faces, if the facial mask is categorized as an indicator of reduced risk or relative safety. Indeed, many studies conducted during the pandemic found that people evaluated masked faces as more positive and trustworthy than unmasked faces, which was also accompanied by a reduced social distancing preference towards masked faces (e.g., Cartaud et al., 2020; Kühne et al., 2022; Lee & Chen, 2021; Marini et al., 2021; Oldmeadow & Koch, 2021; Olivera-La Rosa et al., 2020; but see Fatfouta & Trope, 2022; Seres et al., 2021). In contrast, only two studies reported explicit negative evaluations of masked faces, which included reduced trustworthiness and happiness assignments (Biermann et al., 2021), as well as the judgement that masked persons were more likely ill than unmasked persons (Olivera-La Rosa et al., 2020). While the former result was most pronounced in persons with more negative attitudes towards masks (i.e., persons, who attributed lower protective potential to masks and felt more burdened by wearing them), the latter result emerged in the very early high-risk phase of the pandemic, when masks were relatively new and uncommon in everyday life, and might have been more of a reminder of the current disease threat than of their protective properties. Apart from that, another handful of studies showed that protective face masks compromise the recognition of emotional expressions (e.g., Bani et al., 2021; Carbon, 2020; Fischer et al., 2012; Kastendieck et al., 2022; Kret et al., 2021; but see Calbi et al., 2021) as well as the identification and memorization of faces (e.g. Carragher & Hancock, 2020; Freud et al., 2020). It has been suggested that this impaired recognition of important non-verbal signals that convey a person’s immediate intentions and emotional states may render masked faces more ambiguous and might in turn reduce social trust, resulting in more negative evaluations of the masked person. Yet, this was not directly tested by these previous studies, with the exception of the study by Cartaud et al. (2020), who found the opposite pattern of results (i.e., lower interpersonal distance and higher perception of trustworthiness when target characters were wearing a face mask as compared to no mask). It was therefore rather unlikely that the increased emotional ambiguity of masked faces may have generally increased avoidance of masked faces in the context of the pandemic.

Given the dangers associated with a COVID-19 infection, which is highly contagious, potentially damaging and sometimes even lethal, combined with the low population immunity in early 2021, it was more likely that the unmasked faces would trigger relative behavioral avoidance. This would also be expected based on the psychobiological concept of the “*Behavioral Immune System*” (BIS; Schaller, 2011). The BIS comprises several protective behavioral mechanisms to avoid pathogen infestation, which help to prevent actual infection and its negative consequences. Previous research on the BIS found that people responded with increased disgust to contagion cues, such as skin rashes or sneezes, and quite readily initiated avoidance behavior in situations of increased contagion risk (Gassen et al., 2018; Keller et al., 2022; Mortensen et al., 2010). A virtual reality study further observed that personal and interpersonal spaces were reduced with virtual characters wearing a face mask, especially in persons highly aversive to contagion risks (Geers & Coello, 2022). During the peak waves of the pandemic in 2020 and 2021, unmasked strangers, and even those without visible sickness cues, were most likely perceived as potentially contagious, given that even asymptomatic infections of COVID-19 are easily transmissible (Moghadas et al., 2020). Consequently, during the present study period, which covered the first half of 2021 and included the second COVID-19 wave in Germany, unmasked faces may have promoted behavioral avoidance tendencies as one of the central mechanisms of the BIS, especially in situations in which infectious risks were particularly salient. The explicit formulation of strict mask mandates and social distancing norms during that time should have further intensified this effect. For example, people’s deliberate decision to comply with the new pandemic norms and/or their individual level of fear of contagion may have triggered explicit intentions to particularly avoid contact with unmasked persons. It is currently unclear, however, whether and to what extent such deliberate intentions may affect automatic behavioral tendencies, which are governed by fundamental proxemic behavior norms (e.g., Hall, 1963; Hall et al., 2005; Hayduk, 1978), and have characteristics of rather reflexive behavioral tendencies (e.g., Krieglmeyer et al., 2013).

Altogether, converging evidence from the pandemic suggested more positive deliberate social evaluations of and reduced social distancing tendencies related to masked faces relative to unmasked ones. Further, given the nature of the disease-avoidant psychophysiological mechanisms of the BIS and the strict social distancing norms and mask mandates in Germany during the test period, we expected that our participants would show increased deliberate avoidance of unmasked relative to masked strangers, and particularly so when feeling threatened by the ongoing pandemic. We also explored whether these deliberate avoidance motives would also influence automatic behavioral tendencies. Studies conducted around the same time (Krishna et al., 2021) indeed found that people with low explicit fear of the pandemic, yet more concern about wearing protective masks, exhibited a reduced implicit avoidance bias for unmasked relative to masked faces. At the time of planning and running the studies, we were unable to formulate directed hypotheses, given that the available research provided reasons to expect both, increased approach or increased avoidance tendencies towards masked as compared to unmasked faces. The goal of the current research was, thus, to explore the effect of target masks on deliberate and automatic approach and avoidance behaviors. We additionally implemented two experimental interventions that aimed at manipulating the perception of immediate contagion risk as potential modulators of changes in relative avoidance behavior (i.e., wearing or not wearing a facial mask during the experimental procedure or watching a disease-prime vs. a control-prime video; see Sect. “The present research” for details below), to explore whether increasing or decreasing subjective perceptions of disease threat would moderate potential effects of facial masks on automatic approach vs. avoidance tendencies.

### The present research

We conducted two behavioral experiments intended to further our understanding of the impact of face masks on social interactions in the COVID-19 pandemic. We included both a direct self-report of behavioral intentions and an indirect measure of spontaneous approach and avoidance behavior initiation, thus considering both the level of deliberate decision making in line with the new pandemic social norm, and automatic behavioral tendencies that may rather reflect less deliberate, automatic mechanisms of social cognition (see Radke et al., 2018 for a similar approach). We thereby specifically assessed whether medical-style protective face masks reduced social avoidance relative to unmasked faces in both measures.

We further used two experimental interventions to either reduce or increase the salience of the immediate contagion threat during interactions with unmasked and masked faces. In the first experiment, half of the participants were instructed to wear a protective face mask themselves while performing the online tasks. A mask protects both the wearer and the interaction partner from aerosols and respiratory droplets in close social encounters. Therefore, we expected that the masked participants would exhibit reduced avoidance motives in both the deliberate and the automatic avoidance measures relative to the participants from the unmasked control group. In the second experiment, half of the participants were primed with contagion-related stimuli immediately before they performed each task. For this purpose, we presented an immersive disease video (disease prime), that showed coughing and sneezing people with flu-like symptoms. The disease-primed group was compared to a control group that watched a neutral video with landscape impressions. We hypothesized that the disease prime would create a context of increased immediate contagion risk, that should upturn avoidance motives in the two tasks, and particularly so when dealing with the unmasked faces that would have the highest contagion potential in real life.

We tested two samples of healthy students of various faculties of a North German University in two consecutive online studies during the first half of 2021. In the direct self-report measure, participants reported their decisions to approach or avoid masked or unmasked faces in the hypothetical situation of a street encounter. As the indirect measure, we employed a variant of the Visual Approach/Avoidance by the Self Task (VAAST; Degner et al., 2021; Rougier et al., 2018), in which participants' response latencies were measured for the initiation of approach versus avoidance movement decisions towards masked versus unmasked faces. We additionally assessed participants’ perception of the general disease threat induced by the current pandemic context as a potential predictor of interindividual differences in the approach/avoidance measures. Given the results of Krishna et al. (2021), it seems reasonable to assume that people who felt more threatened by the COVID-19 pandemic would exhibit enhanced social avoidance behavior in response to unmasked faces that signal an increased contagion risk relative to faces carrying a mask.

Both experiments were carried out in accordance with the Declaration of Helsinki and met the requirements of the Ethics Committee of the *Hamburger Ärztekammer* who provided ethical approval. We report how we determined our sample size, all data exclusions (if any), all manipulations, and measures in the study.

*Overall, the present research had three exploratory aims*

- Does masking of target faces affect approach and avoidance behavior?
- Are potential masking effects comparable in direct measures, which should tap rather deliberate behavioral decisions, and indirect measures, which should be more sensitive to automatic behavioral operations?
- Do potential masking effects differ in situations with temporarily increased or decreased salience of disease-threat (as by our manipulations)?

## Experiment 1

### Participants

Participants were recruited from an online job platform of a large German university, local social media student groups, as well as word of mouth. We did not determine minimum sample size a priori, but aimed at testing a minimum of 100 participants over the intended test period of approximately one month, a decision based on practicability (i.e., based on available funding and working hours of research assistants).

**Fig. 1:**
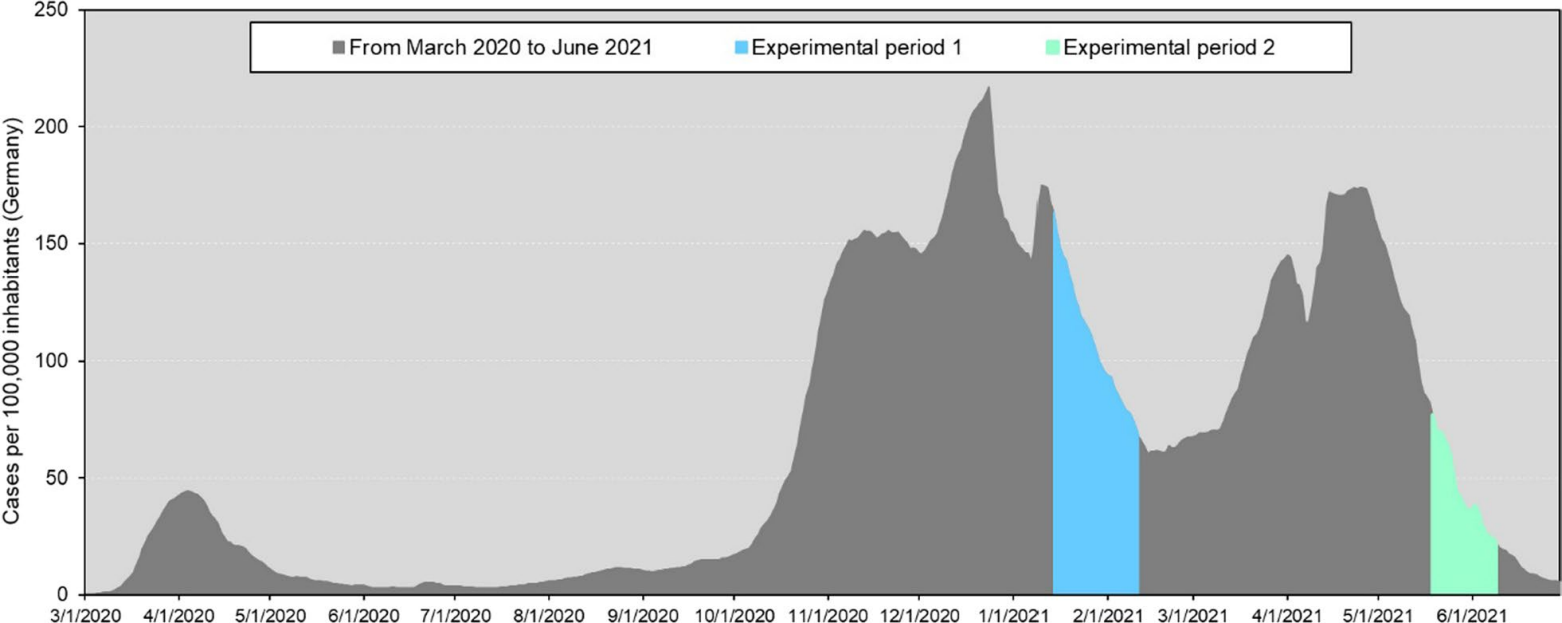
Cases per 100,000 inhabitants in Germany before and during data collection of Experiment 1 and 2. The data for Fig. 1 have been derived from the Robert Koch-Institute (2023)

We included only persons from participation who did not meet the following exclusion criteria: recent or past drug and alcohol abuse, neurological or psychiatric disorders, and chronic physical diseases (e.g., autoimmune diseases). Additional exclusion criteria were prior participation in a similar study within the last few months. Participants provided informed consent and were paid 12€ for participation. The online study took between 35 and 45 min to complete. Experiment 1 was conducted online between the 14th of January and 11th of February, 2021, just following the peak of the second alpha-variant wave of the COVID-19 pandemic in Germany (see Fig. 1). Experiment 1 implemented a between-participants manipulation, aiming at decreasing subjective threat perceptions. Half of the participants were instructed to wear a protective mask while performing the tasks, the other half completed the tasks without wearing a mask. Altogether, we tested 161 people online (median age = 26 years, range 18–42; 108 women and 53 men; participants without a face mask [*control group*]: *n* = 76; 52 women; participants wearing a face mask [*intervention group 1*]: *n* = 84; 55 women). Data of one further participant from the intervention group were excluded from analyses, because the participant indicated in the follow-up interview not having worn a mask during the experiment.

### Stimulus material

This study used face stimuli from "*The dynamics FACES database of emotional expressions in younger, middle-aged, and older adults*" (Ebner et al., 2010). The database contains several pictures and videos of naturalistic faces of women and men from three age groups (young, middle, and old).

**Fig. 2:**
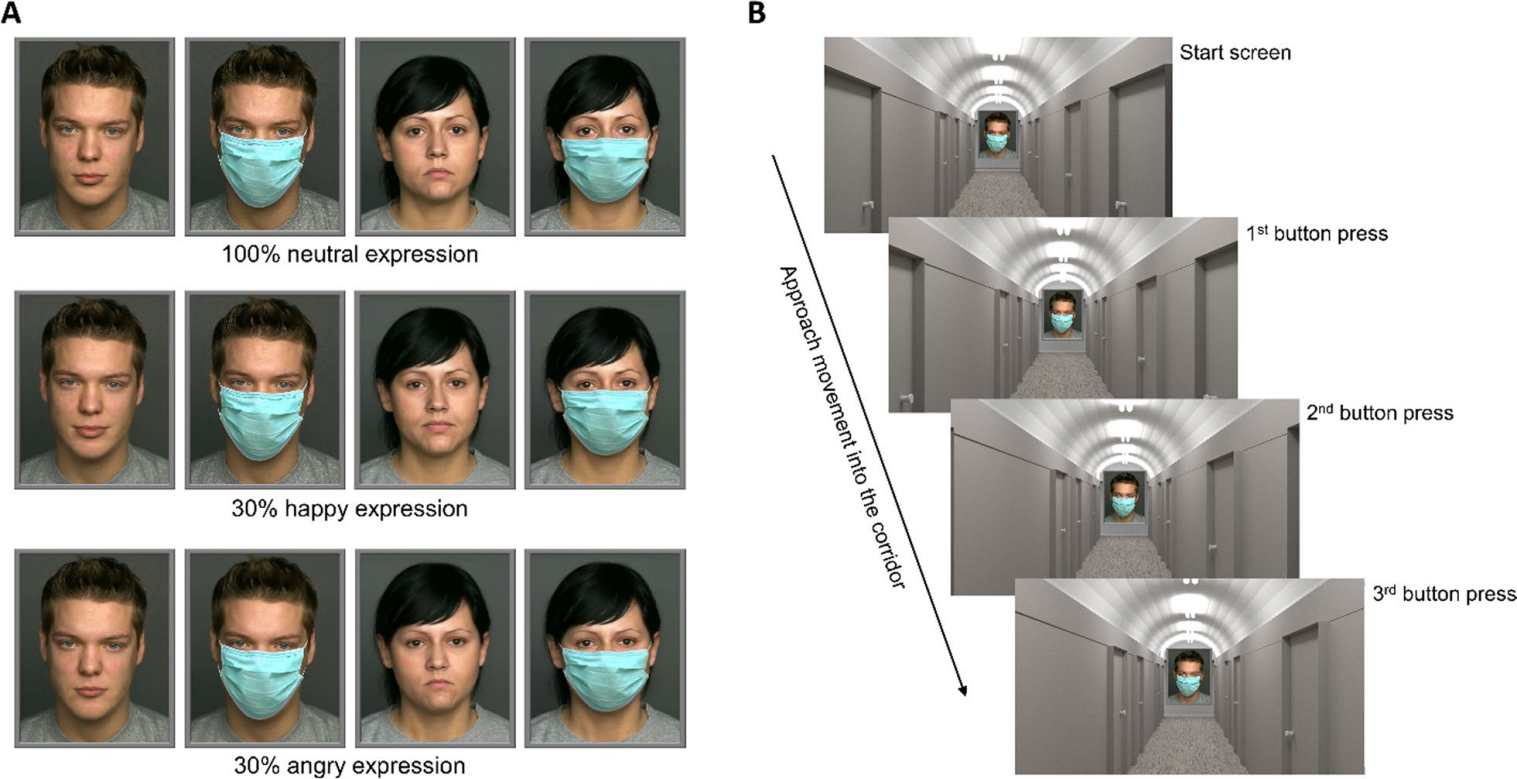
Stimulus material of the direct decision task and the VAAST. (**A**) Exemplary faces of person 140 and 066 from the FACES database (Ebner et al., 2010), with a 100% neutral expression (first row), 30% happy expression (second row), and 30% angry expression (third row). The subtle differences in facial expression were intended to create a more realistic variation in the displayed faces of the direct decision task. Each person was only shown once, either with a mask or unmasked. All participants viewed the same faces in Experiment 1 and 2. It is permitted to show the photos of person 140 and 066 from the FACES database (Ebner et al., 2010) for the purpose of illustrating research methodology. The direct decision task used other persons from the FACES database, who cannot be publicly shown. (**B**) Exemplary VAAST trial, which required an approach movement into the virtual corridor towards the masked face at the opposite wall. The approach was simulated by zooming in by 10% after each button press. The avoidance movement (not shown) was represented by zooming out by 10% from the start screen, thus simulating physical distancing from the face at the opposite wall. All faces shown in the VAAST had a neutral expression

For the direct decision task, we used faces exhibiting subtle expressions of happiness and anger, and also the neutral expressions of women and men between the ages of 18 and 35 years (i.e., from the young sample). The emotions were portrayed by actors in videos in which the facial expression changed from neutral to 100% of the respective emotional expression (i.e., Dynamic FACES; see Fig. 2a). One frame was extracted from the videos as an image displaying 30% emotional intensity, thus looking only very slightly happy or angry. We used these subtle facial expressions in the direct decision task for a more realistic variation in expressions, because in this task participants had no time limitation for answering and could watch the faces for as long as they liked before making the decision to approach or avoid the face. Since all subjects from the two experiments and the different intervention groups received the same set of stimuli, variations in emotional expressions were disregarded in the analyses. Medical face masks were added to half of the images using Adobe Photoshop.

In the VAAST, which required rapid behavioral responses, only neutral facial expressions were shown. The VAAST comprised 10 images from the FACES database (Ebner et al., 2010) showing five young women and five young men with neutral facial expressions. Different from the direct decision task, in the VAAST each person was shown both with and without a face mask.

### Direct approach vs. avoidance decision measure

To assess participants’ deliberate approach vs. avoidance decisions, we presented them with a total of twelve pictures (6 female and 6 male faces) half of which were covered with a medical mask. For each image, participants were asked to provide their behavioral preference while considering the following scenario: “*You meet the person shown below in the street. The person is about 4 m away from your current position. Would you approach her/him (* +*) (e.g., walk closer) or would you rather move away from her/him (-) (e.g., evade the person or even turn around to take a different route)? Please indicate the number of steps you would take towards or away from the person shown.*” The participants rated their personal need for distance on a scale from -4 steps (strong avoidance) to + 4 steps (strong approach). The 0 steps option (no movement) was not available to the participants.

In Experiment 1, the direct decision task was presented through the online survey platform Limesurvey, without response time limit. Before the task, half of the participants received the written instruction to wear a face mask they normally wore in public transport or supermarkets while performing the task. As the experimenter had no direct contact with the participants in the online study, compliance was ascertained by a number of questions regarding mask-related attitudes that were presented after the behavioral tasks on the online platform testable.org. Three subjects from the unmasked group and three from the masked group experienced technical problems and were not able to answer the questions. The remaining participants answered the questions addressing their feelings while wearing a mask during the test session (Q1: *How bothered did you feel by wearing the mask during the test?* Q3: *Did you have the feeling that wearing a face mask during the test impaired your study performance?* Q5: *Did you have the feeling that wearing a mask during the test let you concentrate more on the task and improved your performance?*). The response options to these questions also included the option “*I was not wearing a mask*”, which all, but one participant from the unmasked group, who left this question unanswered, used in response. Apart from that, we also asked for the approximate duration participants had worn the mask during the test, which was on average 29.07 min (SD = 8.70 min) in the masked group, and 0 min in the unmasked group. Eleven persons from the masked group did not indicate the duration, but answered all other questions. We assume that these participants did not keep track of the time, as this was not explicitly requested in advance, and thus failed to provide this information after the test. We also asked all participants on their general feelings when wearing a mask for at least 10 min in public (Q4). This question was answered by all participants, except from the ones who experienced the technical problems. The data of the mask-related questions can be found in OSF (https://osf.io/nv9dz/). We uploaded these data for completeness, but did not further analyze them, since they were simply used to reassure compliance with the intervention.

### Indirect approach vs. avoidance decision measure

We programmed an online variant of the VAAST (Rougier et al. (2018) using Inquisit 6.2.1 web [Computer software] (2020), retrieved from https://www.millisecond.com. In the VAAST, the masked and unmasked faces were displayed against the background of a virtual corridor, with each image hanging on the wall at the end of the corridor (see Fig. 2b). The faces were shown with or without a protective mask. In one block, participants were instructed to approach faces without masks and avoid faces with masks (Block A); in the other block, participants were instructed to approach masked faces and avoid unmasked faces (Block B). The block order was randomly determined in a counterbalanced design, such that half of the participants started the experiment with either Block A or Block B. Each trial started with a gray fixation cross presented for a variable time between 148 and 220ms, which was then replaced by a face. Participants responded to the faces according to the current block’s instruction by pressing the up or down arrow keys three times as quickly and accurately as possible. Depending on the participants' approach or avoidance responses, the whole visual environment was zoomed in by 10% (i.e., approach, press '*walk forward*' up button; see Fig. 2b) or zoomed out (i.e., avoidance, press '*walk backwards*' down button) after each button press, giving the visual impression of moving towards or away from the face. We analyzed only response times (RT) for the first keypress of correct responses.

Participants went through a training phase before the first block, consisting of 10 trials for which they received performance feedback (percentage of correctly completed runs). This was followed by the first experimental block of the main experiment (e.g., Block A). Each experimental block consisted of a total of 40 trials in an individually randomized order. The trial ended 500 ms after the third key press or after an erroneous response.

Prior to analyses, we excluded all trials with incorrect responses as well as individual trials with RTs below 300 ms and above the participant’s arithmetic mean + 2*standard deviations as RT-outliers. Further, we log-transformed response latencies (see Ratcliff, 1993). For ease of interpretation, Tables and Figures provide untransformed values. For facilitation of further analyses, we calculated a VAAST-score as a double difference score by subtracting the mean of compatible trials (approach masked faces, avoid unmasked faces) from incompatible trials (approach unmasked, faces avoid masked faces). While controlling for main effects, a positive (negative) value on this score indicates participant’s response tendency was in line with (contrary to) the expected pattern of taking longer to respond to trials incompatible with preferred behavioral tendencies in a pandemic (approach unmasked, avoid masked) than the compatible ones.

In the first experiment, 43.8% of the participants, who wore a protective face mask, started with Block A and 58% of the unmasked participants started with Block A. Including “*Block sequence*” as an additional factor did not significantly affect results of our analyses and was thus excluded from further analyses.

### Pandemic threat rating and attitude towards masks

After the direct decision task, but before the VAAST, participants rated to what extent they felt threatened by the ongoing COVID-19 pandemic. The question was phrased as “*To what extent do you perceive the corona pandemic as a threat to yourself?*” which participants answered on a 10-point Likert-scale ranging from 1 “*I don’t feel threatened at all*” to 10 “*I feel extremely threatened*”. We also provided the response option “*I don’t know*”, which was set to a missing value in the analyses. However, only two participants from Experiment 1 used this latter response option. We had included three additional questions with regard to participants’ general attitude towards protective face masks, which however had insufficient internal reliability (*α* = 0.438). For the sake of comparability to Experiment 2, which only included the personal threat perception item, we refrain from reporting analyses of all items within this manuscript, but all data and analyses scripts are provided on the Open Science Framework (OSF) for further inspection (https://osf.io/nv9dz/).

### Additional variables

Experiment 1 contained several additional questionnaires regarding social anxiety and distancing preferences, general mask-related attitude as well as an emotion recognition task in the end (see Additional file 1: Table 1). Here, we only analyzed the data from the corresponding tasks and questions of the two experiments. We provide all additional raw data in OSF, but the analysis of these additional variables is out of scope of this paper.

## Experiment 2

### Participants

Recruitment followed the same procedures as in the first experiment. Data collection for Experiment 2 was conducted online between the 19th of May and 9th of June, 2021, when COVID-19 case numbers had again started to decline in Germany (see Fig. 1). Experiment 2 implemented another between-participants manipulation aiming at increasing subjective threat perceptions. Half of the participants were primed with a video showing disease-related content directly before they performed each task. The control group watched a neutral landscape video. The second study was conducted with 150 participants (median age = 24 years, range = *18–35*; 104 women, 45 men, 1 gender-diverse person; landscape video [*control group*]: *n* = 72; 51 women; Disease prime [*intervention group 2*]: *n* = 78; 53 women).

### Stimulus material

The tasks used were the same as those from Experiment 1, except that both the direct decision task and the VAAST were each preceded by either a control or disease prime video. Participants were randomly assigned to one of the video interventions and, different from Experiment 1, they were not requested to wear a mask during the online tests.

### Videos

The videos for the second online study were compiled from various images and short video sequences from the IStock and Pexels databases. Furthermore, excerpts from a video by the Swedish photographer Ulf Lundin (*Bless you*, 1999) were used, in which people were filmed while sneezing. Rights were acquired for images and videos with copyright prior to the study. Each video had a length of 2:15 min. We created two disease prime and two control videos to use directly before the deliberate and the implicit task. This was intended to refresh the influence of the disease prime/control video on the subsequent task, without repeating the same video.

### Approach-avoidance measures

In the second study, the direct decision task and the VAAST were identical to Experiment 1, except that the direct task was implemented within the test protocol of the VAAST programmed and presented in Inquisit Web (6.2.1). In Experiment 2, 53.6% of the participants in the control group started with Block A, whereas 63.6% of the participants primed with the disease video received this block first. Including “*Block sequence*” in the analyses did not significantly affect the results (see analyses scripts in OSF). Therefore, the factor “*Block sequence*” was neglected in all statistical analyses.

### Pandemic threat rating

In Experiment 2, participants also rated to what extent they felt threatened by the ongoing COVID-19 pandemic answering the identical question from Experiment 1. However, different from Experiment 1 the pandemic threat question was asked before the experimental manipulation and the direct decision task and followed several questions referring to self-reports of trait disgust (Olatunji et al., 2007) and trait vulnerability to disease (Duncan et al., 2009). The data from these two inventories will not be further addressed here, as these questionnaires were not included in Experiment 1. They are available in OSF for further inspection. Note that due to this minor change in test sequence and the exposure to additional disgust- and disease-related questions might have affected participants’ responses in the pandemic threat ratings.

### Additional variables

Similar to Experiment 1, this experiment also included additional variables (i.e., disgust and disease vulnerability related inventories) that did not correspond to those of the initial experiment (see Additional file 1: Table 2). Here, we only analyzed the data from the corresponding tasks and questions of the two experiments. We provide all raw data in OSF, but the analysis of these additional variables is out of scope of this paper.

## Results of Experiment 1

### Pandemic threat rating

Participants, who were wearing a mask during testing, exhibited a significantly higher threat rating than the control group not wearing a protective mask, indicating that the priming manipulation increased the salience of infectious threat, *t*(157) = 2.023, *p* = .045, *d* = 0.321, 95%CI [0.008; 0.634].

### Direct approach vs. avoidance decision measure

**Fig. 3:**
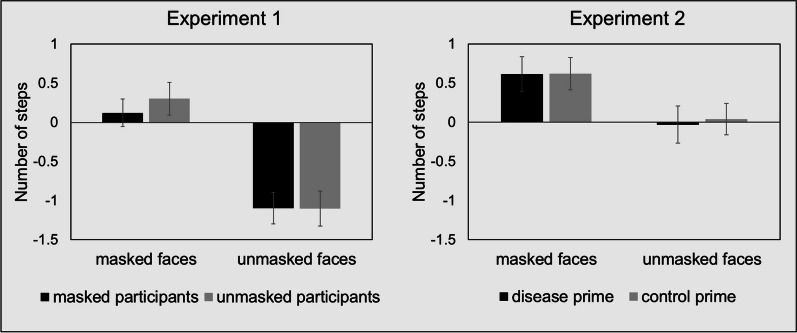
Deliberate approach vs. avoidance intentions in social interactions in relation to protective face masks (Experiment 1 and 2). Figure shows mean number of steps per participant group and target face condition and standard errors of the mean

The two (participant masking: masked vs. unmasked) by two (target face: masked vs. unmasked) analyses of variance of self-report approach vs. avoidance tendencies indicated a significant main effect of target face, *F*(1, 158) = 67.420, *p* < .001, *η*_*p*_^2^ = .299, 90% CI [.000; .948], and yielded no significant effects of participant masking, with *F*(1, 158) = 0.133, *p* = .716, *η*_*p*_^2^ < .001, 90% CI [.000; .0035] for the main effect, and *F*(1, 158) = 0.338, *p* = .562, *η*_*p*_^2^ = .002, 90% CI [.000; .083] for the interaction. Thus, independent of themselves wearing a mask, participants generally expressed a stronger intention to approach a masked face (*M* = .208, SD = 1.727) than an unmasked face (*M* = − 1.099, SD = 1.879; *t*(159) = 8.209, *p* < .001, *d* = .649, 95% CI [.478; .819]; see Fig. 3).

**Fig. 4:**
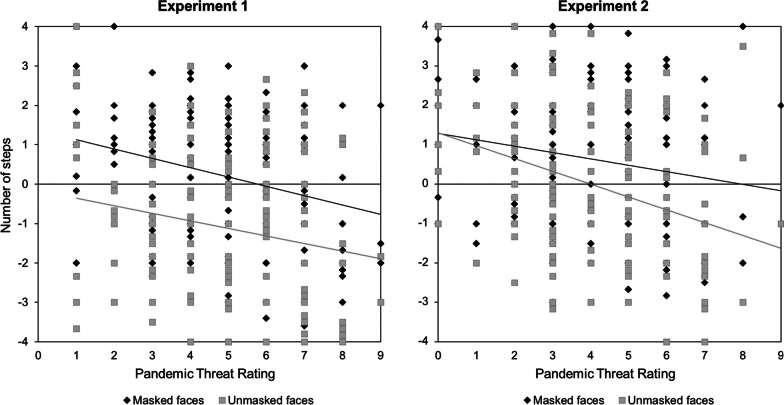
The subjective perception of pandemic threat, as determined by the question: “*To what extent do you perceive the corona pandemic as a threat to yourself?*”, correlated with increased deliberate social distancing from both masked and unmasked faces in both experiments

Deliberate approach-avoidance decisions were significantly related to participants’ subjective pandemic-related threat perceptions for both masked faces, *r*(159) = − .269, *p* < .001, 95% CI [− .408; − .119], and unmasked faces, *r*(159) = − .200, *p* = .016, 95% CI [− .345; − .046]. We additionally computed a deliberate behavior score (i.e., the difference score of response decisions for masked minus non-masked faces), indicating interindividual differences in participants relative tendency to avoid unmasked compared to masked faces. This score was however not significantly related to participants’ subjective pandemic-related threat perceptions, *r*(159) =  − .044, *p* = .580, 95% CI [− .198; .112]. Participants who judged the COVID-19 pandemic higher in personal threat, tended to avoid faces in general more than participants with lower threat perceptions, regardless of whether these faces were masked or not (see Fig. 4).

### Indirect approach vs. avoidance decision measure (VAAST)

The two (participant masking: masked vs. unmasked) by two (target face: masked vs. unmasked) by two (movement: approach vs. avoid) analysis of variance of log-transformed response latencies in the VAAST yielded a significant main effect of movement, *F*(1,158) = 19.171, *p* < .001, *η*_*p*_^2^ = .108, 90% CI [.000; .838], and target face, *F*(1, 158) = 29.827, *p* < .001, *η*_*p*_^2^ = .159, 90% CI [.000; .889]. Importantly, we observed no significant two-way interaction between target face and movement, *F*(1, 158) = .003, *p* = .958, *η*_*p*_^2^ < .001, 90% CI [.000; .001], nor a significant three-way interaction involving all factors, *F*(1, 158) = .744, *p* = .390, *η*_*p*_^2^ = .005, 90% CI [.000; .167]. Thus, participants’ automatic behavior was generally faster for approach decisions (*M* = 559 ms, SD = 146 ms) than avoidance decisions (*M* = 577 ms, SD = 208 ms; *t*(159) = 4.383, *p* < .001, *d* = .347, 95% CI [.186; .506], and faster when responding to masked faces (*M* = 563 ms, *SD* = 185 ms) than unmasked faces, (*M* = 573 ms, SD = 164 ms; *t*(159) = 5.453, *p* < .001, *d* = .431, 95% CI [.268; .592], see Table 1. For correlational analyses, we calculated a double difference score of log-transformed response times in the VAAST representing the interaction of masked vs. unmasked faces with approach vs. avoidance movements. This score was neither significantly correlated with the direct measure of behavioral intentions, *r*(160) = .022, *p* = .779, 95% CI [− .133; .177], nor with participants’ threat perceptions *r*(159) = − .021, *p* = .793, 95% CI [− .176; .135].

**Table 1:** Means (in ms) of untransformed response latencies in the VAAST in Experiments 1 and 2 (Standard deviations in parentheses)

	Intervention groups		Control groups	
Experiment 1	Masked participants		Unmasked participants	
	Masked target	Unmasked target	Masked target	Unmasked target
Approach	548 (112)	555 (109)	565 (252)	572 (146)
Avoid	555 (111)	567 (98)	568 (98)	600 (326)

Experiment 2	Disease prime video		Control prime video	
	Masked target	Unmasked target	Masked target	Unmasked target
Approach	517 (107)	522 (93)	503 (94)	514 (90)
Avoid	525 (88)	533 (83)	518 (85)	529 (84)

## Results of Experiment 2

### Pandemic threat rating

Participants in the disease prime group exhibited a significantly higher threat rating than the control group of Experiment 1, *t*(148) = 2.235, *p* = .027, *d* = .365, 95% CI [.042; .688]. This was insofar surprising, as the question referring to the subjective pandemic threat perception was asked before the randomly assigned disease vs. control video priming manipulation. As a potential confound this higher rating could have thus amplified the effect of the disease prime on subsequent behavior. Yet, we found that the disease prime video neither influenced deliberate avoidance decisions nor automatic approach/ avoidance behavior in the group comparisons (see below).

### Direct approach vs. avoidance decision measure

The two (video prime: disease vs. control) by two (target face: masked vs. unmasked) analysis of variance of self-report approach vs. avoidance tendencies replicated the significant main effect of target face observed in Experiment 1, *F*(1, 148) = 23.252, *p* < .001, *η*_*p*_^2^ = .136, 90% CI [.000; .870], and yielded no significant effects of video prime, *F*(1, 148) = .020, *p* = .889, *η*_*p*_^2^ < .001, 90% CI [.000; .006] for the main priming effect, and *F*(1, 148) = .065, *p* = .798, *η*_*p*_^2^ < .001, 90% CI [.000; .018] for the interaction. Thus, independent of the salience of the disease prime, participants generally expressed a stronger intention to approach a masked than an unmasked face, *t*(149) = 4.851, *p* < .001, *d* = .396, 95% CI [.229; .562] (see Fig. 3).

Deliberate approach-avoidance decisions were significantly related to participants’ subjective pandemic threat perceptions for both masked faces, *r*(150) = − .200, *p* = .014, 95 %CI [− .349; − .041], and unmasked faces, *r*(150) = − .352, *p* < .001, 95% CI [− .485; − .203]. Additionally, the deliberate behavior score (i.e., the difference score of response decisions for masked minus non-masked faces) indicating interindividual differences in participants relative tendency to avoid unmasked compared to masked faces was also significantly related to participants’ subjective pandemic threat perceptions, *r*(150) = .198, *p* = .015, 95% CI [.039; .347]. Participants who judged the COVID-19 pandemic higher in personal threat, tended to avoid faces in general, but more strongly avoided unmasked faces as compared to masked faces than participants with lower threat perceptions.

### Indirect approach vs. avoidance decision measure (VAAST)

The two (video prime: disease vs. control) by two (target face: masked vs. unmasked) by two (movement: approach vs. avoid) analysis of variance of log-transformed response latencies in the VAAST tendencies yielded a significant main effect of movement, *F*(1, 148) = 28.654, *p* < .001, *η*_*p*_^2^ = .162, 90% CI [.000; .892], and target face, *F*(1, 148) = 25.609, *p* < .001, *η*_*p*_^2^ = .148, 90% CI [.000; .880]. Importantly, again, we observed no significant two-way interaction between target face and movement, *F*(1, 148) = 0.002, *p* = .962, *η*_*p*_^2^ < .001, 90% CI [.000; .001], nor a significant three-way interaction involving all factors, *F*(1, 148) = .065, *p* = .798, *η*_*p*_^2^ < .001, 90% CI [.000; .018]. Thus, participants’ automatic behavior was generally faster for approach than avoidance decisions, *t*(151) = 4.741, *p* < .001, *d* = .385, 95% CI [.219; .549], and faster when responding to masked vs. unmasked faces, *t*(151) = 4.403, *p* < .001, *d* = .357, 95% CI [.193; .521], see Table 1. Again, we calculated a double difference score of log-transformed response times in the VAAST representing the interaction of masked vs. unmasked faces with approach vs. avoidance movements. This score was neither significantly correlated with the direct measure of behavioral intentions, *r*(151) = − .0194, *p* = .815, 95% CI [− .178; .141], nor with participants’ threat perceptions *r*(151) = .031, *p* = .707, 95% CI [− .130; .190].

## Comparison of the control samples from Experiments 1 and 2

Experiments 1 and 2 were conducted consecutively during a quickly evolving global pandemic with tremendous changes in case numbers, social restrictions, policy debates, and public access to vaccination. For example, COVID-19 case numbers per 100,000 German inhabitants significantly differed between the studies, with case numbers being more than 2.5 times higher at the time of Experiment 1 than 2 (*M*_*1*_ = 110.0, SE = 2.0 cases; *M* _*2*_= 38.2, SE = 1.4 cases), while vaccinations remained widely unavailable for the younger German population at both time periods (Bundesregierung, 2023). It is generally possible that these differences in pandemic ramifications affected social avoidance in the two samples independent of the interventions. For this reason, we combined data from both experiments to compare the quantifiable aspects of pandemic ramifications and avoidance behavior in the two control samples.

We indeed observed significantly higher pandemic threat ratings in the participants of Experiment 1 (unmasked control group: *M* = 4.58, SD = 1.91; masked group: *M* = 5.20, SD = 1.99) relative to Experiment 2 (group with control video: *M* = 3.69, SD = 2.04; disease prime group: *M* = 4.41, *SD* = 1.88), when directly comparing the control groups, *t*(146) = 2.727, *p* = .007,* d* = .449 95% CI [.121; .774]) and the intervention groups, *t*(159) = 2.602, *p* = .010,* d* = .410 95% CI [.097; .722], of both experiments.

When including Experiment as an additional between-subjects factor in the analyses of the direct decision task, we observed a significant interaction between target faces (masked vs. unmasked) and Experiment (1 vs. 2), *F *(1,311) = 11.124, *p* < .001, *η*_*p*_^2^ = .035, 90% CI [.000; .605]. As can be seen in Fig. 3, there was a marginal but significantly stronger approach tendency towards masked faces in Experiment 1 than in Experiment 2, *t*(311) = 1.969, *p* = .050, *d* = .223, 95% CI [.001; .445]. Reversely, there was a stronger avoidance tendency towards unmasked faces in Experiment 1 than in Experiment 2, *t*(311) = 4.998, *p* < .001, *d* = .565, 95% CI [.338; .791].

Replicating the relative robustness of automatic approach and avoidance behavior in the VAAST, we observed no significant main effect or interactions in relation to Experiment as can be verified with the data files provided in OSF.

## Discussion

Our research investigated deliberate and automatic social avoidance tendencies towards masked and unmasked faces in healthy young participants, who were tested following two major waves of the COVID-19 pandemic in 2021. The analyses were based on two independent online samples, who received different interventions to either reduce or enhance the immediate disease threat by the presented stimuli.

Participants In the direct approach vs. avoidance decision task showed a generally higher willingness to approach a masked as opposed to an unmasked person in both experiments. Additionally, in Experiment 1 we also observed an increased avoidance preference related to the unmasked faces. These effects were not affected by manipulations intended to increase the salience of infectious diseases, such as wearing a mask during participation in Experiment 1 or watching videos of sneezing individuals in Experiment 2. Additionally, participants’ deliberate behavioral tendencies were significantly related to their subjective perceptions of personal risks that the pandemic poses.

No such effects were observed with regard to automatic behavioral tendencies measured with the VAAST, where behavioral decisions were always faster for approach than avoidance decisions, regardless of whether targets were wearing masks or not, regardless of our experimental manipulations of disease salience, and regardless of participants subjective threat perceptions of the pandemic.

Overall, the present data suggest that the absence of protective face masks may have led to increased social avoidance at the level of deliberate decision making during the ongoing pandemic with enhanced contagion threat. Yet, automatic social avoidance was rather unaffected by the new pandemic norm to keep an interpersonal distance to unmasked faces.

### Reduced social approach/increased avoidance preferences for unmasked faces in the direct decision task

In both experiments, hypothetical interaction partners were more readily approached when they were wearing a protective mask compared to being unmasked. Moreover, the negative mean values in Experiment 1 indicated that the unmasked faces were in fact deliberately avoided, whereas in Experiment 2, participants rather showed reduced approach, as reflected by a small, yet positive mean value. This difference was also confirmed in the direct comparison of the control groups of both experiments. Taken together, these observations are in line with previous findings that showed a reduced social distancing preference to masked virtual agents (Cartaud et al., 2020). This may supposedly reflect a more positive attitude towards masked faces as suggested by other studies from the COVID-19 pandemic (e.g., Oldmeadow & Koch, 2021), and may also conform with the homeostatic theory of social interactions, which posits that appropriate social distance depends on the peripersonal space representation combined with the emotional valence of the social stimulus (Coello & Cartaud, 2021). As the latter probably increased when the social interaction partner was wearing a mask (Cartaud et al., 2020), the selected social distance may have been shorter or behavioral approach may have even been prioritized. In early 2021, case numbers were high in Germany and significantly higher than during Experiment 2 (RKI, 2021). Further, strict social distancing measures were still in effect, while vaccinations were unavailable for the younger German population (Bundesregierung, 2023), rendering our student participants more vulnerable to contract a severe COVID-19 infection. In combination with the generally increased pandemic threat ratings of Experiment 1, it seems plausible to assume that the second wave of the alpha-variant of the coronavirus may have led to an increased social distancing preference, which probably triggered particularly strong deliberate avoidance motives in Experiment 1.

Moreover, when considering the interindividual differences in pandemic threat perception, we found a relation between the subjective threat rating and increased deliberate avoidance in both experiments. This suggests that, if participants felt personally threatened by the ongoing pandemic, they deliberately decided to keep a larger physical distance from other humans. In Experiment 2, we further observed that higher threat ratings were also associated with higher deliberate behavior scores, indicating that masked faces were more readily approached than unmasked ones by individuals who felt more threatened by the pandemic. This also fits with the results of a previous study, in which participants who associated protective masks with the threat of the coronavirus, felt socially more distant to unmasked than masked faces (Grundmann et al., 2021).

### No effects of protective face masks on automatic social avoidance behavior

So far only two other studies addressed the influence of protective face masks on automatic social avoidance behavior. In their pre-print, Ingram et al. (2021) report that a large online sample from the UK (*n* = 622) and the US (*n* = 619), recruited via Prolific Academic (Palan & Schitter, 2018), was quicker to approach masked than unmasked faces in the VAAST, and also evaluated the masked faces as more trustworthy. However, Ingram et al. (2021) found no differences in avoidance latency. Krishna et al. (2021) used the VAAST in two English-speaking international online samples of young (*n* = 147) and elderly persons (*n* = 150), also recruited online with Prolific. Their study was performed between January and February 2021, and thus during a similar time frame as our Experiment 1. Krishna et al. (2021) found no general behavioral bias in the samples. Yet, they found that at least a subgroup of people, who were less concerned by the pandemic, but more worried about wearing a mask, exhibited a reduced avoidance bias for unmasked relative to masked faces (Krishna et al., 2021). In our studies, however, we did not observe any effect of masks on the speed of approach vs. avoidance decisions in the VAAST. The measure was neither sensitive to our experimental manipulations nor to interindividual differences in perceptions of pandemic threat. Finally, approach and avoidance tendencies in our direct and indirect measures were unrelated to each other. The inconsistencies in results between these and our studies indicate that effects of masks on automatic social approach behavior may not be very robust or potentially moderated by unknown contextual factors. For example, the samples assessed by the three studies differed in regional origin. We tested two online samples from Northern Germany, who experienced the same regional pandemic measures and societal debates during their test periods. In contrast, Krishna et al. (2021) assessed an international sample consisting of English-speaking people from very different countries, such as Canada, UK, South Africa, Italy, and Portugal. Moreover, Ingram et al. (2021) tested two large samples of people from the UK and the US. This population diversity and the regional variation in lockdown measures, media coverage of the pandemic and societal debates, as well as in the actual mortality rates and COVID-19 case rates in the different countries could have thus contributed to differences in automatic avoidance behavior.

Altogether, our observations suggest a top-down modulation of deliberate decisions by the new pandemic norm that mandated social distancing from (unmasked) strangers. Apparently, this deliberate behavioral motive was not transferred to the level of the automatic behavioral tendencies, as demonstrated by the data from two consecutive experiments. Future replication studies will still have to reassess these findings and account for the different contextual effects on automatic avoidance. For example, one may investigate to what extend societal debates about wearing face masks (including very different levels of ideological polarization) may have influenced internalization of social distancing norms among participants from different countries and thus differentially affected approach avoidance behaviors. Additionally, it may be possible that alterations in automatic avoidance behavior only slowly follow the rather abrupt changes in deliberate behavior and might thus only consistently emerge after several months or even years, provided that the contextual factors have also been altered permanently. However, since COVID-19 has lost most of its threatening potential (e.g., population immunity has significantly increased since 2021 and the dominant variants-of-concern have become endemic) and mask mandates have been dropped completely, it is very unlikely to find any changes in the automatic behavioral motives related to masked faces in the near future.

## Conclusion and future directions

This research project assessed deliberate and automatic social avoidance of masked and unmasked faces during the COVID-19 pandemic. The deliberate decision for increased social distancing from strangers in a hypothetical encounter was clearly influenced by protective face masks, resulting in an increased willingness to approach a masked as opposed to an unmasked person in both experiments. This suggests that the absence of protective face masks may have increased relative social avoidance, when considered on the explicit level of deliberate decision making. In contrast, automatic avoidance motives, as measured by the VAAST, were not affected by protective measures. Since previous and our own study assessed the influence of protective face masks on social decision making and behavior during the COVID-19 pandemic, it currently remains an open question whether the observed effects will remain stable once the coronavirus has become endemic and protective measures such as masks are no longer required or whether they will also disappear in deliberate decisions. Future studies thus have to carefully reassess deliberate and automatic behavioral motives towards masked and unmasked faces in the post-pandemic period.

### Supplementary Information

**Additional file 1**. Sequence of tasks and questionnaires in the two studies.

## Data Availability

All data generated or analyzed in this study are provided on OSF (https://osf.io/nv9dz/).
