# Supplementary material for: The COVID-19 pandemic and changes in social behavior: Protective face masks reduce deliberate social distancing preferences while leaving automatic avoidance behavior unaffected
Source: Cogn Res Princ Implic. 2024 Jan 8;9:2. doi: 10.1186/s41235-023-00528-4 (PMC10772029; doi:10.1186/s41235-023-00528-4)
Supplement: Supplementary file 1 — Additional file 1. Sequence of tasks and questionnaires in the two studies. [file 41235_2023_528_MOESM1_ESM.pdf]

Table 1: Sequence of tasks and questionnaires of online study 1.

Sequence	Type	Brief summary	Number of items/ trials	Included in both studies
**1.**	**Demographic questions**	**Questions on age and sex** (+ other demographics)	**6**	**Both studies (questions on age and sex)**
**2.**	**Explicit decision Task**	**Deliberate approach avoidance of masked and unmasked faces**	**12**	**Both studies**
3.	*Selected social anxiety questions*	*Questions from the German SASKO trait inventory (Kolbeck & Maß, 2009) (Items: 10, 12, 21, 23, 25, 27,31, reversed 12)*	*8*	*Only study 1*
**4.**	**Pandemic threat question (Corona 1)**	**Question on the subjective feeling of threat elicited by the pandemic**	**1**	**Both studies**
5.	*Questions on subjective feelings related to the pandemic*	*Additional questions addressing personal relations and current social distancing behavior in the pandemic*	*4*	*Only study 1*
6.	*Questions addressing general mask-related attitudes*	*Questions related to the importance and acceptance of mask-mandates*	*3*	*Only study 1*
7.	*Social distance rating*	*Three questions regarding the currently preferred distance from loved ones, acquaintances or strangers*	*3*	*Only study 1*
8.	*Comments*	*Free comments*	*1*	*Only study 1*
**9.**	**VAAST**	**Implicit approach avoidance task with masked and unmasked faces**	**40**	**Both studies**
10.	*Emotion recognition task*	*Basic emotion recognition in masked and unmasked faces (anger, disgust, happy, neutral)*	*48*	*Only study 1*
**11.**	**Mask-related questions (feelings during experiment)**	**Questions related to wearing a mask during the experiment**	**5**	**Only study 1 (but of interest for the analysis)**

Tasks and questionnaires included in both studies are marked in bold font.

Table 2: Sequence of tasks and questionnaires of online study 2.

Sequence	Type	Brief summary	Number of items/ trials	Included in both studies
**1.**	**Demographic questions**	**Questions on age and sex**	**2**	**Both studies**
2.	*State-related questions*	*Questions related to current health state and stress*	*9*	*Only study 2*
3.	*Selected autoritarism questions*	*Questions from the KSA-3 (Beierlein et al., 2014)*	*4*	*Only study 2*
4.	*DS-R Disgust trait questionnaire*	*Items from the core disgust and contamination disgust scales of the DS-R (Olatunji et al., 2007)*	*17*	*Only study 2*
5.	*Vulnerability of Disease trait questionnaire*	*Complete questionnaire (germ aversion and perceived infectability scales)(Duncan et al., 2009)*	*15*	*Only study 2*
**6.**	**Pandemic threat question (Corona 1)**	**Question on the subjective feeling of threat elicited by the pandemic**	**1**	**Both studies**
7.	*Video prime (Part 1)*	*Disease or control video prime*	*1*	*Only study 2*
**8.**	**Explicit decision Task**	**Deliberate approach avoidance of masked and unmasked faces**	**12**	**Both studies**
9.	*Video prime (Part 2)*	*Video prime from same category as video 1, yet showing different humans or landscape impressions (Part 2)*	*1*	*Only study 2*
**10.**	**VAAST**	**Implicit approach avoidance task with masked and unmasked faces**	**40**	**Both studies**
11.	*Selected state questions from the MDES questionnaire*	*Items from the German modified differential emotions scale (MDES) (amusement, disgust, surprise, shame) (Brandenburg & Backhaus, 2015)*	*4*	*Only study 2*
12.	*Relative state feelings*	*Questions on feelings relative to the time before the test (stressed, inspired, sick, uncomfortable, optimistic, amused)*	*6*	*Only study 2*
13.	*Attention and perceived realness questions*	*Subjective evaluation of the attention to the videos as well as the perceived realness of the video content*	*8*	*Only study 2*

Tasks and questionnaires included in both studies are marked in bold font.

References

Kolbeck S, Maß R (2009) Fragebogen zu sozialer Angst und sozialen Kompetenzdefiziten. 1. Auflage, Hogrefe-Verlag.

Beierlein C, Asbrock F, Kauff M, Schmidt P (2014) Ein ökonomisches Messinstrument zur Erfassung dreier Subdimensionen autoritärer Einstellungen. GESIS Working Paper 35.

Olatunji BO, Williams NL, Tolin DF, Abramowitz JS, Sawchuk CN, Lohr JM, Elwood LS (2007) The Disgust Scale: item analysis, factor structure, and suggestions for refinement. Psychol. Assess. 19, 281.

Duncan LA, Schaller M, Park JH (2009) Perceived vulnerability to disease: Development and validation of a 15-item self-report instrument. Personal. Individ. Differ. 47, 541–546.

Brandenburg S, Backhaus N (2015) Zur Entwicklung einer deutschen Version der modified Differential Emotions Scale (mDES). Conference Paper: 11. Berliner Werkstatt Mensch-Maschine-Systeme.
